# Study of the binding of ΔFN3.1 fragments of the *Bifidobacterium longum* GT15 with TNFα and prevalence of domain-containing proteins in groups of bacteria of the human gut microbiota

**DOI:** 10.20517/mrr.2023.06

**Published:** 2023-04-12

**Authors:** Maria G. Alekseeva, Ilya N. Dyakov, Kristina K. Bushkova, Dilara A. Mavletova, Roman A. Yunes, Irina N. Chernyshova, Ilya A. Masalitin, Tatiana A. Koshenko, Venera Z. Nezametdinova, Valery N. Danilenko

**Affiliations:** ^1^Laboratory of Genetics of Microorganisms, Vavilov Institute of General Genetics, Russian Academy of Sciences, Moscow 119991, Russia.; ^2^Laboratory of Immunoglobulin biosynthesis, Mechnikov Research Institute of Vaccines and Sera, Moscow 105064, Russia.; ^3^Caspian International School of Medicine, Caspian University, Almaty 050000, Kazakhstan.

**Keywords:** Fibronectin domain type III (FN3), bifidobacteria, TNFα, host-bacteria interaction

## Abstract

**Aim:** This study is mainly devoted to determining the ability of ∆FN3.1 protein fragments of *Bifidobacterium* (*B.*) *longum* subsp. *longum* GT15, namely two FN3 domains (2D FN3) and a C-terminal domain (CD FN3), to bind to tumor necrosis factor-alpha (TNF-α).

**Methods:** Fragments of the *fn3* gene encoding the 2D FN3 and CD FN3 were cloned in *Escherichia* (*E.*) *coli*. In order to assess the binding specificity between 2D FN3 and CD FN3 to TNFα, we employed the previously developed sandwich ELISA system to detect any specific interactions between the purified protein and any of the studied cytokines. The trRosetta software was used to build 3D models of the ∆FN3.1, 2D FN3, and CD FN3 proteins. The detection of polymorphism in the amino acid sequences of the studied proteins and the analysis of human gut-derived bacterial proteins carrying FN3 domains were performed *in silico*.

**Results:** We experimentally showed that neither 2D FN3 nor CD FN3 alone can bind to TNFα. Prediction of the 3D structures of ΔFN3.1, 2D FN3, and CD FN3 suggested that only ΔFN3.1 can form a pocket allowing binding with TNFα to occur. Polymorphism analysis of amino acid sequences of ΔFN3.1 proteins in *B. longum* strains uncovered substitutions that can alter the conformation of the spatial structure of the ΔFN3.1 protein. We also analyzed human gut-derived bacterial proteins harboring FN3 domains which allowed us to differentiate between those containing motifs of cytokine receptors (MCRs) in their FN3 domains and those lacking them.

**Conclusion:** Only the complete ∆FN3.1 protein can selectively bind to TNFα. Analysis of 3D models of the 2D FN3, CD FN3, and ΔFN3.1 proteins showed that only the ΔFN3.1 protein is potentially capable of forming a pocket allowing TNFα binding to occur. Only FN3 domains containing MCRs exhibited sequence homology with FN3 domains of human proteins.

## INTRODUCTION

Type III fibronectin domains (FNIII, FN3) were first identified in eukaryotic fibronectin^[1-3]^, where they function as structural spacers or mediate protein-protein interactions^[4]^. The FN3 domain contains 90-100 amino acid residues, which form a conserved beta (β)-sandwich fold. The fold of the FN3 module contains seven strands, forming two antiparallel β-sheets of three and four strands, which are stacked on top of each other, forming a hydrophobic core that does not require stabilization by intrachain disulfide bonds^[1]^.

**Figure 1:**
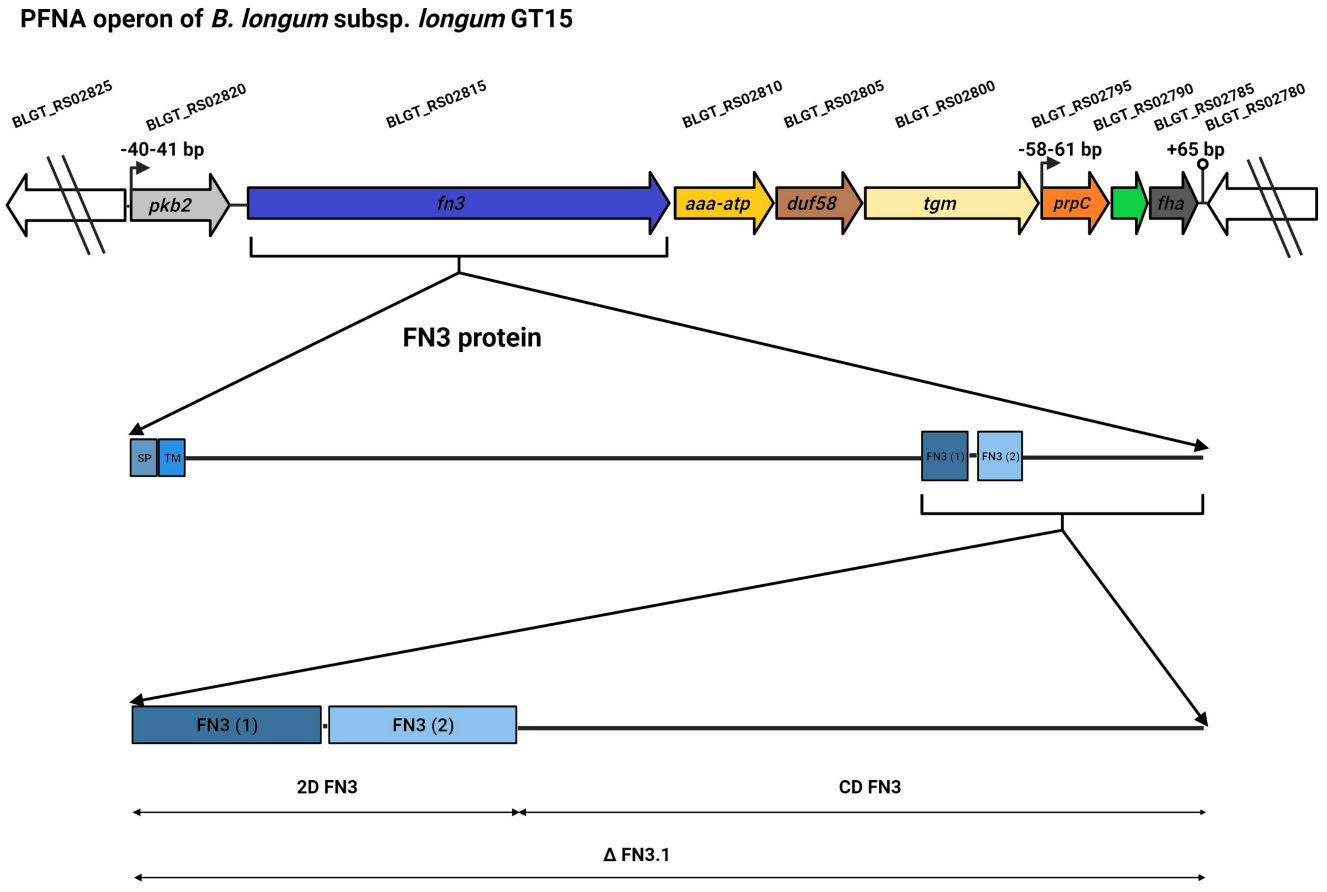
Diagram illustrating the PFNA operon of *B. longum* subsp. *longum* GT15, and the FN3 protein and its fragments (∆FN3.1 protein, 2D FN3, CD FN3) used in our experiments aimed at testing the protein’s ability to bind the cytokine TNFα. The genes of the PFNA operon are *pkb2* (serine-threonine protein kinase Pkb2, BLGT_RS02820, AIW43409.1), *fn3* (fibronectin type III do-main-containing protein, BLGT_RS02815, AIW43408.1), *aaa-atp* (AAA-ATPase MoxR, BLGT_RS02810, AIW43407.1), *duf58* (hypothetical protein with DUF58 domain, BLGT_RS02805, AIW43406.1), *tgm* (transglutaminase, BLGT_RS02800, AIW43405.1), *prpC* (protein phosphatase, BLGT_RS02795, AIW43404.1), hypothetical protein (BLGT_RS02790, AIW43403.1), *fha* (FHA do-main-containing protein, BLGT_RS02785, AIW43402.1). SP: signal peptide; TM: transmembrane.

The FN3 domain is widespread among proteins of both eukaryotic organisms such as humans, animals, plants and fungi and prokaryotic organisms such as bacteria, archaea and viruses. FN3 domains have been found in various eukaryotic extracellular and intracellular protein families, including extracellular matrix molecules, enzymes, muscle tissue proteins, and cell surface receptors [including motifs of cytokine receptors (MCRs)]^[5,6]^. FN3 domains often contain the consensus motif WSXWS (WS motif; MCR; random amino acid in the middle of the motif)^[7,8]^. This motif plays a role in receptor folding, ligand (cytokine) binding, and signal transduction^[9,10]^. Mutations in this motif alter the receptor’s ability to bind cytokines^[8]^. In bacteria, FN3 domains are found in proteins with different enzymatic functions: cellulases^[11,12]^, hydrolases^[1,13]^, chitinases^[14,15]^, and proteases^[16]^. Presumably, FN3 domains play a structural, stabilizing role in ensuring the normal functioning of the enzyme^[12]^. FN3 domains have been shown to be evolutionarily conserved and involved in a wide range of cellular functions: cell adhesion, migration, growth and differentiation^[17,18]^, the transmission of nerve impulses^[19,20]^ and biofilm formation^[21]^. Researchers based at the Laboratory of Microbial Genetics of the Vavilov Institute of General Genetics were the first to discover the unique and species-specific PFNA operon and to shed light on its role in communication with the human immune system via interaction with cytokines^[22,23]^. The PFNA operon was found in the genomes of 77 out of 99 species of *Bifidobacterium*, including 11 species that are commonly found in humans. The PFNA operon consists of five to eight genes depending on the concrete species of *Bifidobacterium*^[23]^. The main genes are *pkb2*, *fn3*, *aaa-atp*, *duf58* and *tgm*. The structure of the PFNA operon of *Bifidobacterium* (*B.*) *longum* subsp. *longum* GT15, which consists of eight genes, is illustrated in Figure 1.

The serine-threonine protein kinase Pkb2, encoded by the *pkb2* gene, is a signal transduction protein capable of binding to an external signal; the ligand is currently not known. The *fn3* gene encodes the FN3 protein, which contains two fibronectin domains that have MCRs [Figure 1]. This protein has a signal peptide and a transmembrane domain. It is found only in *Bifidobacterium*. We demonstrated for the first time that a fragment of the FN3 protein, which consists of two FN3 domains and a C-terminal region (∆FN3.1; Figure 1), can selectively bind to the tumor necrosis factor TNFα^[24]^. The studied AAA+ ATPase of the MoxR Proper subfamily encoded by the *aaa-atp* gene is a chaperone that is actively phosphorylated by the protein kinase Pkb2^[22]^. It was shown that the cultivation of the strain *B. longum* subsp. *longum* GT15 in the presence of TNFα leads to a significant increase in the expression of genes making up the PFNA operon^[25]^.

**Figure 2:**
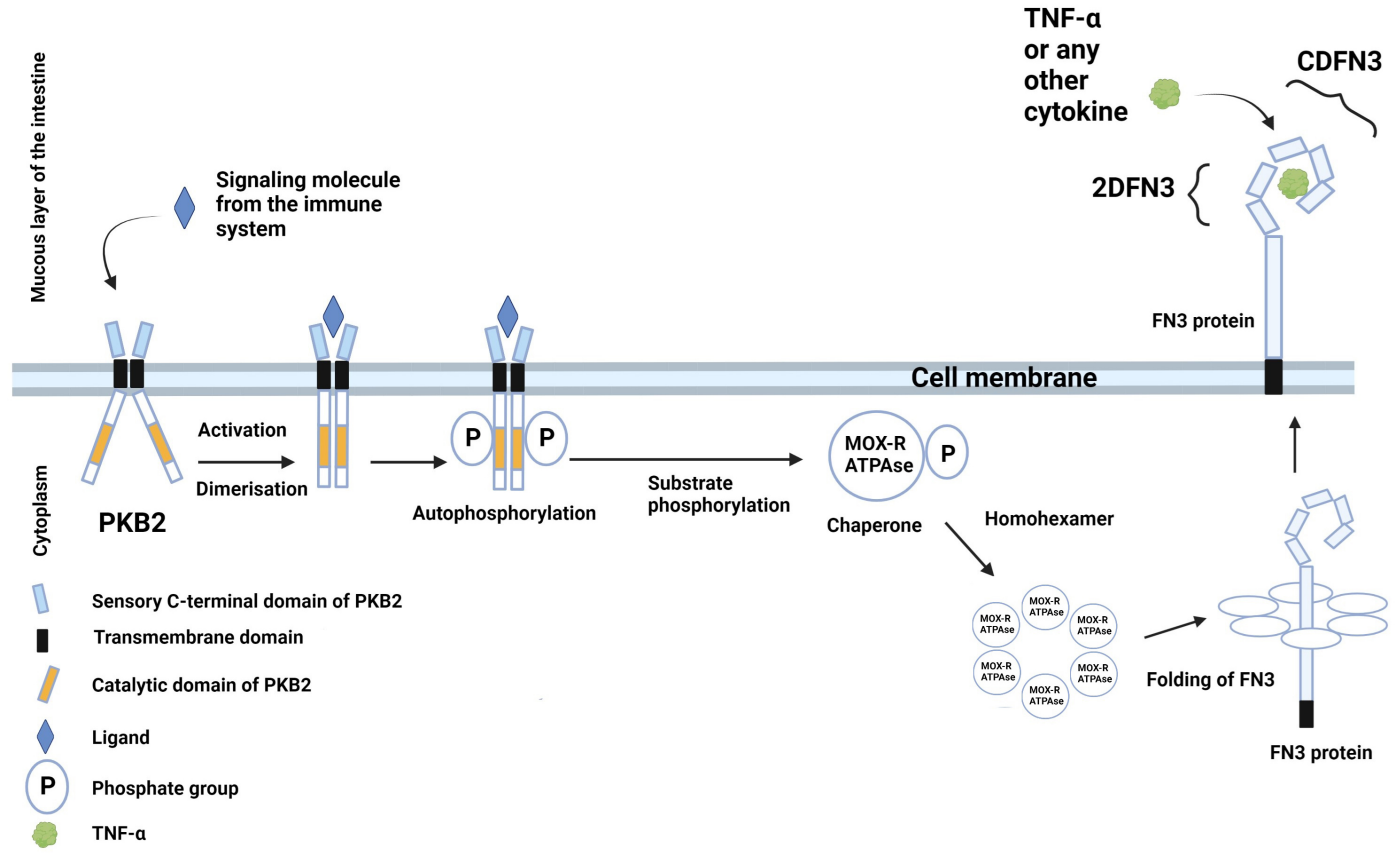
Hypothetical schematic illustration of the interaction between proteins encoded by the PFNA operon of *B. longum* subsp. *longum* GT15 and elements of the host’s immune system. The Pkb2 signaling system is activated by an unknown ligand, which is most likely a component of the host’s immune system (possibly TNFα). Serine-threonine protein kinase Pkb2, following its activation by a ligand, autophosphorylated^[22,23]^ and phosphorylated other substrates, including the MoxR-ATPase protein encoded by the *aaa-atp* gene of the PFNA operon^[22]^. MoxR-ATPase is a chaperone that is possibly involved in the folding of proteins, including the FN3 protein. FN3 is a secreted protein possessing a transmembrane domain that is presumably attached to the outer surface of the bacterial cell membrane. It has been experimentally shown that the ΔFN3.1 protein is capable of selective binding of the cytokine TNFα^[24]^. The strain *B. longum* subsp. *longum* GT15 also increases the expression of TNFα, IL8, and IL10 cytokines in human cells^[27]^. Apparently, TNFα interacts with an unknown signaling system in *B. longum* subsp. *longum* GT15 (possibly, Pkb2), upregulating the expression of the PFNA operon genes, including the *fn3* gene encoding the FN3 protein^[25]^, which is capable of binding TNFα^[24]^. Figure 2 offers a putative mechanism of bidirectional interaction between *Bifidobacterium* and the human immune system.

Previously, we proposed a hypothetical scheme of interaction between proteins encoded by the PFNA operon and the host immune system [Figure 2]^[22,26]^.

The present work is part of the study of the mechanism of interaction between *Bifidobacterium* and elements of the host’s immune system and the role of the ∆FN3.1 protein fragment encoded by the unique species-specific PFNA operon in this process. The most important part of the presented study is devoted to determining the ability of fragments of the ∆FN3.1 protein [Figure 1] separately, namely the two FN3 domains (2D FN3) and the C-terminal domain (CD FN3), to bind the tumor necrosis factor TNFα. The spatial structures of the studied protein fragments were also predicted to identify potential cytokine-binding regions. In this study, we performed a bioinformatic analysis of the presence of FN3 domains in proteins of bacteria of the genus *Bifidobacterium*, as well as the families *Lactobacillaceae*, *Bacteroidaceae*, and *Clostridioides*, which are typical inhabitants of the human intestinal microbiota in order to detect proteins potentially capable of interacting with elements of the host’s immune system.

## METHODS

### Bacterial strains, plasmid vectors, culture media and conditions

In our study, we used the strains: *B. longum* subsp. *longum* GT15^[28]^, *Escherichia* (*E.*) *coli* DH5a (F^-^, Ф 80 ΔlacZΔM15, Δ(lacZYA-argF), U169) (Promega, USA)^[29]^ and *E. coli* BL21(DE3) [F^-^, dcm, ompT, hsdS(rB^-^mB^-^), gal λ (DE3)] (Novagen, USA) and the expression vector pET16b (Novagen, USA)^[30]^ containing a His-Tag linker in the N-terminal region for protein isolation and purification.

The model strain *B. longum* subsp. *longum* GT15 was used. *B. longum* subsp. *longum* GT15 belongs to the most studied and widespread species: *B. longum*. This strain was isolated from the feces of a healthy adult in our laboratory and deposited in the Russian National Collection of Industrial Microorganisms [VKPM, Moscow, Russia, http://eng.genetika.ru/service-offer/vkpm/; VKPM is a member of the World Federation for Culture Collections (WFCC) and European Culture Collections’ Organization (ECCO)]. The international catalog number in VKPM: VKPM AC-1928. The genome of *B. longum* subsp. *longum* GT15 was sequenced (status complete, NZ_CP006741.1)^[31]^. We conducted RNA-Seq analysis^[25,32]^. This strain is the main object of molecular genetic research in our laboratory. It is also a component of the probiotic preparation “Genobact”. The strain *B. longum* subsp. *longum* GT15 was cultivated anaerobically (HiAnaerobic SystemeMark III, HiMedia, India) in agar and MRS broth (HiMedia, India) supplemented with cysteine (0.5 g/L) at 37 °C for 24-48 h. *E. coli* strains were cultivated in Luria-Bertani (LB) broth^[31]^. Ampicillin (150 μg/mL) was used as a selective marker for cells containing plasmids.

### DNA manipulations

The genomic DNA of *B. longum* subsp. *longum* GT15 was isolated using the GenElute Bacterial Genomic DNA Kit (Sigma-Aldrich, USA). Isolation of plasmid DNA, obtaining competent *E. coli* cells, transformation and analysis of recombinant plasmids were performed using standard methods^[31]^.

Fragments of the *fn3* gene encoding the 2 FN3 domains and encoding the C-terminal region were amplified from the strain *B. longum* subsp. *longum* GT15 genomic DNA using the Phusion High-Fidelity PCR Master Mix kit (Thermo Fisher Scientific, Lithuania) on a PTC-0150 MiniCycler (MJ Research, Inc., USA). A fragment of the *fn3* gene reaching 537 bp in length encoding two domains of the FN3 domain (stretching from nucleotide 4,480 to nucleotide 5,013) was amplified using the oligonucleotides FN3-N (^5´^tcgtcatatgcccgagccgccactgctc^3´^) and FN3-CD (^5´^gatcctcgagctaggactcggcactccaattga^3´^). The fragment encoding the C-terminal region (stretching from nucleotide 5014 to nucleotide 5985; overall fragment size including the stop codon = 972 bp) was amplified using FN3-CN (^5´^tcgtcatatggccgcatcgccgtcagaga^3´^) and FN3-C (^5´^gatcctcgagctactgcttgtgaatggtggt^3´^) oligonucleotides. The resulting fragments were cloned between the *Nde*I and *Xho*I restriction sites in the pET16b expression vector containing His-Tag for protein isolation and purification. The sequences of all cloned fragments were confirmed by the sequencing of the corresponding plasmid DNA.

### Expression of the cloned genes in E. coli and purification of recombinant 2D FN3 and CD FN3 proteins

*E. coli* BL21(DE3) cells harboring recombinant plasmids containing the cloned genes were first grown in LB broth at 37 °C to an optical density of 0.6-0.8 (~2 h). Gene expression was induced by adding 1.0 mM isopropyl-β-D-thiogalactoside (IPTG). Next, the cells were cultured at 25 °C for 18 h, after which they were pelleted by centrifugation (5,000 rpm, 10 min, 4 °C) and stored at -20 °C. To study the expression of the cloned genes, cells were suspended in sample buffer (pH 6.8) containing 62.5 mM Tris-HCl, 5% glycerol, 2% 2-mercaptoethanol, 0.1% SDS, 0.001% and bromophenol blue. Then, the cells were lysed by heating them to 95 °C for 10 min and were analyzed using SDS-PAGE afterwards. *E. coli* BL21(DE3) protein fractions containing the pET16b plasmid without an insert were used as a negative control.

Protein isolation and purification was carried out following the method described by us earlier^[24]^. For further protein purification, dialysis was employed in PBS buffer containing 10% glycerol and 1 mM PMSF. The concentration of the isolated protein was measured on a Qubit 2.0 fluorometer (Invitrogen, USA). Purified proteins were stored at -80 °C.

### Assessment of the ability of FN3 protein fragments to bind to human TNFα

In our study, we used polyclonal rabbit IgG antibodies specific to the FN3 protein. The techniques of production, extraction and purification of antibodies were described by us earlier^[24]^. In the devised ELISA test described previously^[24]^, anti-FN3 antibodies were used as a “trap” for the FN3 protein. The polyclonal nature of these antibodies allowed us to assume that they also cross-react with 2D FN3 and CD FN3 fragments, which was verified from the outset of the study.

Nunc MaxiSorp 96-well polystyrene plates were sensitized with recombinant FN3, 2D FN3 fragment and CD FN3 fragment at a concentration of 5 µg/mL in 100 µL carbonate-bicarbonate buffer (CBC) (18 mM Na_2_CO_3_, 450 mM NaHCO_3_, pH 9.5) for one h at 37 °C. The plates were subsequently washed four times with phosphate-buffered saline with 0.05% Tween 20 (PBST). In order to exclude nonspecific adsorption, 1% casein was added to the wells and incubated for 1 h at 37 °C. Next, 100 μL of polyclonal rabbit IgG antibodies was added to the wells in two-fold dilutions starting from a concentration of 1 mg/mL, incubated at 37 °C for one h and washed four times with PBST. To exclude nonspecific binding between rabbit IgG and FN3 and its fragments, nonspecific rabbit γ-globulin was added to FN3 instead of polyclonal rabbit IgG antibodies. Goat anti-rabbit IgG horseradish peroxidase (HRP) conjugate was added to the wells at a 1:20,000 dilution. The plates were incubated for 60 min at 37 °C, washed four times with PBST, and 3,3'5,5'-tetramethylbenzidine was added to them. The reactions were stopped with 1 M sulfuric acid. Optical density was measured at a wavelength of 450 nm using a Universal Microplate Reader (Biotek, Winooski, VT, USA). The results were treated statistically with an unpaired t-test.

After confirming the binding of polyclonal rabbit IgG antibodies to the whole FN3 protein including its 2D FN3 and CD FN3 fragments, their ability to bind human TNFα was assessed. The whole FN3 protein was used as a positive control. To do this, we used the previously devised ELISA scheme^[24]^. Below is a brief description of it.

Nunc MaxiSorp 96-well polystyrene plates were sensitized with 100 µl polyclonal rabbit IgG antibodies to the FN3 protein in concentration 1 µg/mL, incubated in carbonate-bicarbonate coating buffer (CBB) for 1 h at 37 °C, followed by washing with the phosphate-buffered solution with 0.05% Tween 20 (PBST). In order to exclude nonspecific adsorption, 1% casein was added to the wells and incubated for 1 h at 37 °C. After washing, 100 µL of the FN3 protein solution, 2D FN3 or CD FN3 fragments at a concentration of 2 µg/ml were added to the wells, incubated for 1 h at 37 °C, and washed 4 times with PBS. In the following step, a cytokine solution was added at a concentration of 250 pg/ml, incubated for 1 h at 37 °C, and washed as described above. Finally, the HRP conjugate of IgG to TNFα (CYTOKINE, Russia) was added to the wells. Incubation, washing and color reaction using 3,3'5,5'-tetramethylbenzidine were carried out as described previously^[24]^.

All the obtained data were subjected to statistical analysis using the GraphPad Prism 6 software and the nonparametric Mann-Whitney test.

### Bioinformatics analysis

Bioinformatics analysis of the occurrence of proteins with FN3 domains in the sequenced genomes of bacteria belonging to the genus *Bifidobacterium* and the families *Lactobacillaceae*, *Bacteroidaceae*, *Clostridioides* isolated from the human gastrointestinal tract was carried out using the NCBI database (http://www.ncbi.nlm.nih.gov/). Only proteins with annotated functions were used in the analysis.

The following programs were used in this study: BLAST (http://blast.ncbi.nlm.nih.gov/Blast.cgi) for detection of amino acid polymorphisms; Clustal Omega (http://www.ebi.ac.uk/Tools/msa/clustalo/) for multiple amino acid sequence alignments; SMART (http://smart.embl-heidelberg.de/) for identification of FN3 protein domains; UniProt (http://www.uniprot.org/) for identification of signal peptides and transmembrane domains; SAS (https://www.ebi.ac.uk/thornton-srv/databases/sas/) to identify homology with 3D structures of proteins presented in the Protein Data Bank (PDB) database; trRosetta (https://yanglab.nankai.edu.cn/trRosetta/) for predicting the spatial structures of proteins^[33]^.

## RESULTS

### Study of the binding of fragments of the FN3 protein of the strain B. longum subsp. longum GT15 to TNFα

The FN3 protein encoded by the *fn3* gene of the PFNA operon of *B. longum* subsp. *longum* GT15 consists of 1,994 amino acid residues and harbors two FN3 domains, which stretch from amino acid 1,494 to amino acid 1,581 and from amino acid 1,586 to amino acid 1,671, respectively. The region following the second FN3 domain (from amino acid 1,672 to amino acid 1,994) is the C-terminal region of the protein. Previously, we cloned in *E. coli* a fragment of the *fn3* gene encoding the part of the protein stretching from amino acid 1,494 to amino acid 1,994 and encompassing both the FN3 domains and the C-terminal region. After obtaining the recombinant protein (designated by us as ΔFN3.1), we were able to demonstrate that this fragment of the FN3 protein can selectively bind to the tumor necrosis factor TNFα^[24]^.

In this study, we tested the ability of the fragments that make up the ΔFN3.1 protein: 2D FN3 (from amino acids 1,494 to 1,671; containing only two FN3 domains) and CD FN3 (from amino acids 1,672 to 1,994; containing only the C-terminal region of the protein) to binding to TNFα. The molecular weights of the recombinant proteins corresponded to the calculated molecular weights (including the pET16b plasmid linker containing His-Tag) of the 2D FN3 protein fragments stretching from amino acid 1494 to amino acid 1,671 (21 kDa) and CD FN3 stretching from amino acid 1,672 to amino acid 1,994 (36 kDa).

At the next stage of the work, the possibility of purification of recombinant 2D FN3 and CD FN3 proteins was tested under conditions that were previously optimized for the ∆FN3.1 protein^[24]^. The recombinant 2D FN3 and CD FN3 proteins [Supplementary Figure 1] were isolated and purified in sufficient quantities to study their binding to TNFα.

At the first stage of the study, cross-linking between polyclonal rabbit IgG antibodies and the FN3 protein harboring 2D FN3 and CD FN3 fragments was established. The process of obtaining antibodies was described in our previous paper^[24]^. We established that polyclonal rabbit IgG antibodies were able to bind to the mentioned fragments at high dilutions - a positive reaction was detected for both 2D FN3 and CD FN3 at a concentration of 0.008 μg/mL. For the sake of comparison, the reaction between ΔFN3.1 and antibodies was detected previously at a concentration of 0.004 μg/mL. The addition of nonspecific γ-globulins instead of polyclonal antibodies did not lead to a cross-reaction with FN3, nor with its fragments.

**Figure 3:**
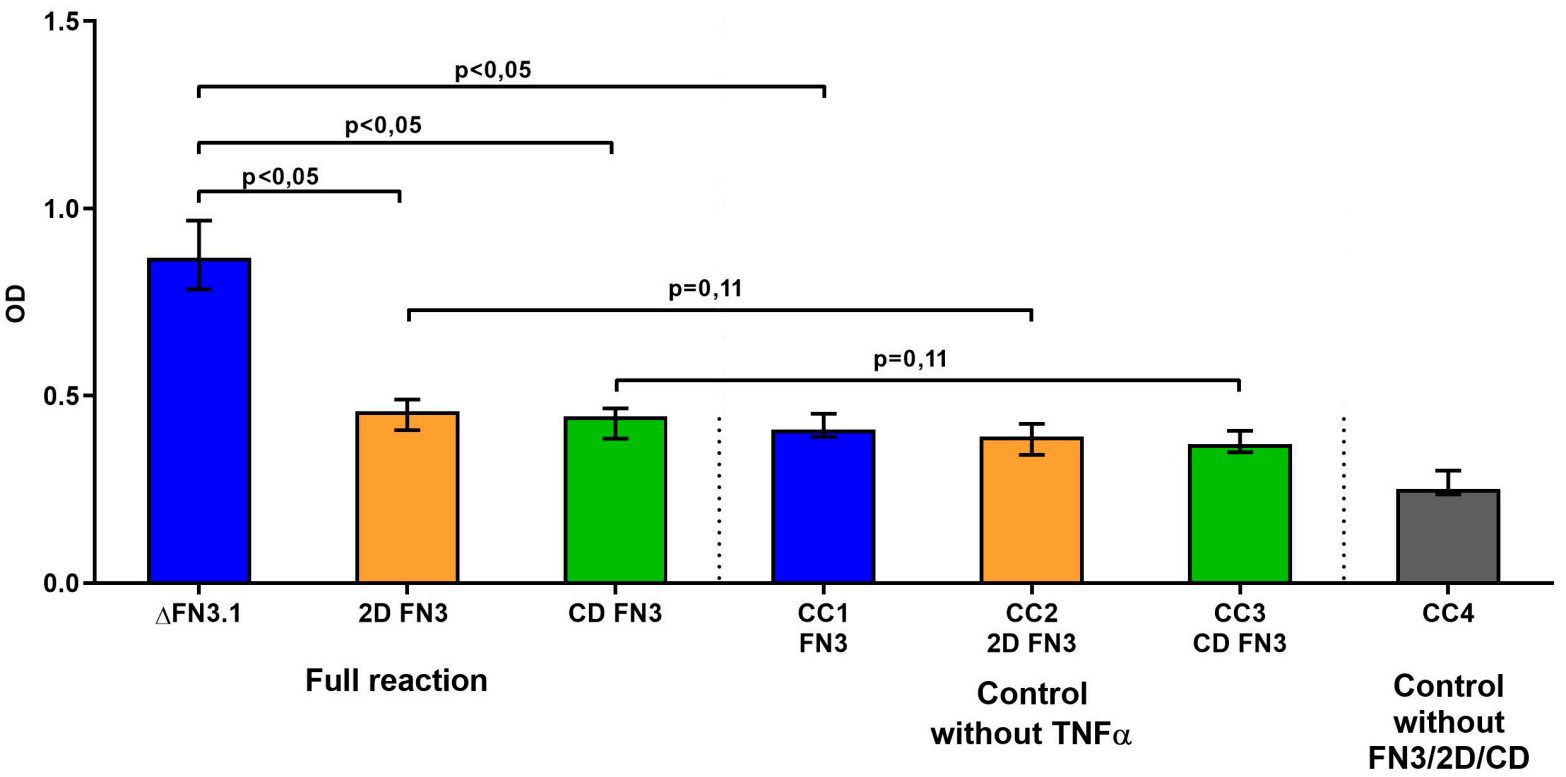
Specific interaction of the whole ΔFN3.1 protein, consisting of both 2D FN3 and CD FN3 fragments, with TNFα. CC1-CC4 - «conjugate control» (without TNFα for each protein or without proteins - ΔFN3.1, 2D FN3 or CD FN3).

In order to assess the binding specificity between 2D FN3 and CD FN3 to TNFα, we employed the ELISA scheme described above and in our previous publication^[24]^. First, FN3-specific polyclonal rabbit IgG antibodies were sensitized on the solid phase. Second, 2D FN3 or CD FN3 fragments or the whole FN3 protein was added as a “second layer”. Next, a solution of TNFα conjugated to HRP from the CYTOKINE commercial kit (Russia) was added. The devised ELISA scheme is a well-validated model for the detection of interaction between FN3 and TNFα. However, the results showed that neither 2D FN3 nor CD FN3 bound TNFα [Figure 3]. Our data indicate that for binding to TNFα, the complete fragment ΔFN3.1 containing both 2D FN3 and CD FN3 fragment is necessary.

### Prediction of 3D structures of FN3 protein fragments of B. longum subsp. longum GT15

**Figure 4:**
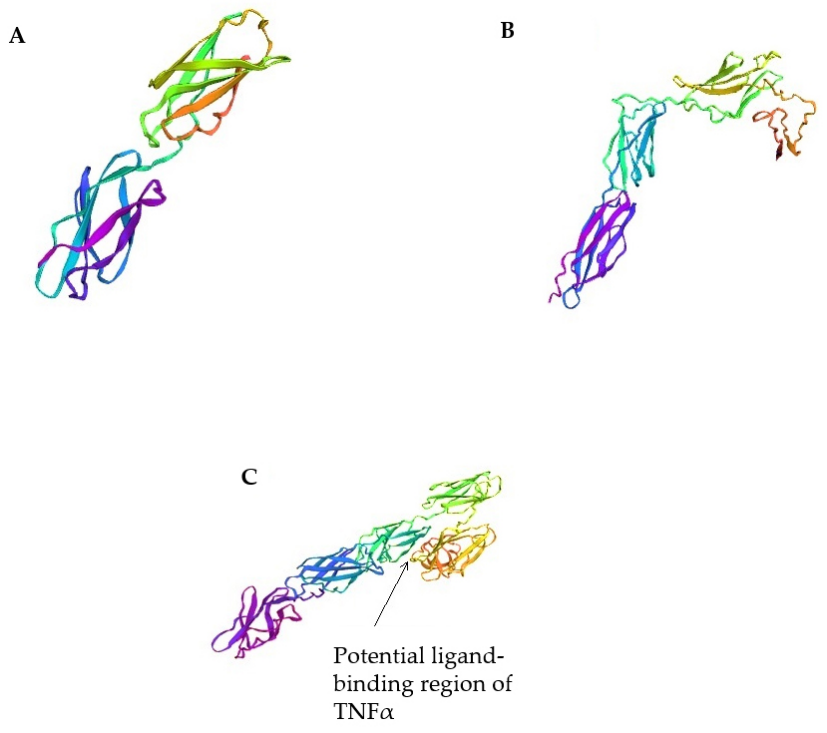
Three-dimensional models of fragments of FN3 protein of the strain *B. longum* subsp. *longum* GT15 using the trRosetta servers: (A) 2D FN3 protein; (B) CD FN3 protein; (C) ΔFN3.1 protein.

Since the 3D structures of bifidobacteria proteins containing FN3 domains have not yet been determined, we used the trRosetta servers to predict the spatial structures of the studied proteins. In this study, using trRosetta software, we built structural models of fragments of FN3 proteins of the strain *B. longum* subsp. *longum* GT15: 2D FN3 [Figure 4A], CD FN3 [Figure 4B], and ΔFN3.1 [Figure 4C] proteins, with a prediction confidence of 0.710, 0.506, and 0.527, respectively.

Three-dimensional models of 2D FN3 and ΔFN3.1 proteins were constructed on the basis of homologous modeling using previously obtained crystal structures of eukaryotic proteins containing FN3 domains from PDB: crystal structure of the full ectodomain of human Gp130 (PDB code 3L5H), human Ifnw-Ifnar ternary complex (code PDB 3SE4), structure of a cytokine receptor complex (PDB code 4NKQ), and crystal structure of PTPdelta ectodomain in complex with IL1RAPL1 (PDB code 4YH7). The 2D FN3 protein model is formed by two linear FN3 domains dominated by antiparallel β-sheets [Figure 4A]. The ΔFN3.1 protein model includes five structural elements (epitopes), with three structural elements that tightly interact with each other forming a V-shaped block [Figure 4C]. We conjecture that the binding between the ΔFN3.1 protein of the *B. longum* GT15 strain and TNFα or other cytokines occurs specifically in this region (pocket). Since no homologous structures were found for the CD FN3 protein fragment (C-terminal region), a 3D model was built de novo. This model is formed by three structural elements, which are also dominated by antiparallel β-layers [Figure 4B].

### Polymorphisms of amino acid sequences of FN3 protein fragments of B. longum strains, the impact of identified substitutions on predicted 3D structures

Previously, the comparative analysis of sequences encoding the regions 2D FN3 in the sequenced genomes of *B. longum* subsp. *longum* allowed us to divide them into four groups^[26]^. The revealed polymorphism of amino acid sequences of ΔFN3.1 proteins in 203 sequenced genomes of *B. longum* subsp. *longum* also made it possible to distinguish four groups of strains [Supplementary Figure 2]. Group 1 encompassed all strains harboring identical amino acid sequences of ΔFN3.1, including that of the strain GT15 and was considered as a reference. As for group 2, the strains contained a single substitution: 43 A→V. Strains belonging to group 3 contained four substitutions: 43 A→V, 51 A→T, 417 P→Q and 424 A→T. Strains belonging to group 4 contained three substitutions: 111 T→I, 417 P→Q and 424 A→T.

**Figure 5:**
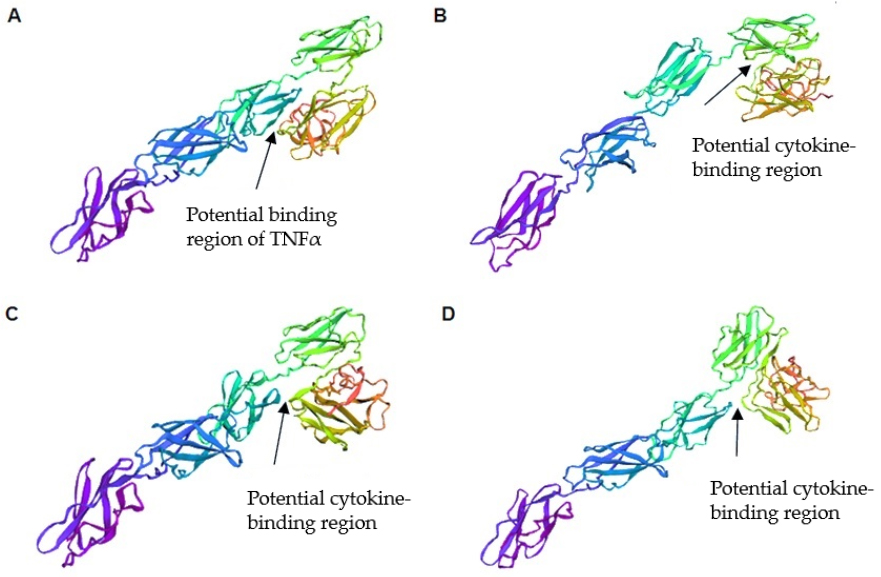
Three-dimensional models of ΔFN3.1 proteins from different groups of *B. longum* subsp. *longum* using the trRosetta server: (A) group 1 (do not contain amino acid substitutions in comparison to the reference strain *B. longum* GT15); (B) group 2 (contained a single substitution 43 A→V); (C) group 3 (contained four substitutions 43 A→V, 51 A→T, 417 P→Q and 424 A→T); (D) group 4 (contained three substitutions: 111 T→I, 417 P→Q and 424 A→T).

Using trRosetta software, we predicted the spatial structures of ΔFN3.1 proteins from all four groups of *B. longum* subsp. *longum* strains [Figure 5].

The amino acid substitutions detected in ΔFN3.1 proteins lead to conformational changes in structural elements of the predicted protein models. Significant differences were observed in the region of three structural elements that form a V-shaped block, in which binding to cytokines can occur. Thus, amino acid differences in the sequences of ΔFN3.1 proteins from four groups of *B. longum* subsp. *longum* can account for the specificity of binding to cytokines.

### Bioinformatics analysis of the occurrence of proteins containing FN3 domains in commensal bacteria inhabiting the human intestinal microbiota

Proteins containing fibronectin domains carry out diverse functions and are widespread among all bacteria, including commensal members of the human and animal intestinal microbiota. Previously, we identified and characterized the species-specific PFNA operon, which was found only in the genus *Bifidobacterium*. One of the key genes of the cluster is the *fn3* gene, which encodes a protein harboring two type 3 fibronectin domains (FN3) containing MCRs^[22,23]^.

Bioinformatics analysis of proteins with FN3 domains in the sequenced genomes of bifidobacterial strains isolated from the human gastrointestinal tract showed that, apart from the FN3 domain encoded by the PFNA operon, most bifidobacterial species also contain proteins annotated as glycosyl hydrolase (GH) family 3 (beta-glucosidase); these proteins typically contain in the central or C-terminal regions an FN3 domain lacking MCRs. *B. dentium* strains each contained 12 proteins belonging to the GH family 3 [Supplementary Table 1].

The PFNA operon is found only in bifidobacteria. Other bacteria (including commensal bacteria of the human gastrointestinal tract) contain proteins that carry FN3 domains. Only the FN3 domains containing annotated MCRs exhibit similarities with the FN3 domains of human proteins. Our bioinformatics analysis of the sequenced genomes of bacterial strains belonging to the families *Lactobacillaceae*, *Bacteroidaceae*, and *Clostridioides* revealed the presence of a number of proteins containing a single FN3 domain, most often in the C-terminal or the central region of the protein. We were able to divide the identified proteins into two groups: Group 1: proteins containing annotated MCRs in the FN3 domains [Supplementary Table 2]; and Group 2: proteins lacking MCRs in the FN3 domains [Supplementary Table 3].

Group 1 encompassed GHs, which represent a common group of enzymes (EC 3.2.1.) that hydrolyze the glycosidic bond between two or more carbohydrates or between a carbohydrate and a non-carbohydrate moiety. Bacteria of the *Lactobacillaceae* and *Bacteroidaceae* families contained proteins belonging to the GH family 31 (alpha-glucosidase and alpha-xylosidase) containing a single FN3 domain each, in which MCRs were found. Bacteria belonging to *Bacteroidaceae* possessed proteins of the GH family 43 (1,4-beta-xylanase), while bacteria belonging to *Clostridioides* contained proteins of the GH family 18 and family 20b (hyaluronoglucosaminidase). 

Proteins lacking MCRs in FN3 domains were assigned to Group 2. Proteins originating from *Bacteroidaceae* belonged to the GH family 20 (beta-hexosaminidase) and to the GH family 92 (alpha-mannosidase) and proteins of *Clostridoides* belonged to the GH family 65 (maltose phosphorylase). Bacterial proteins originating from the *Lactobacillaceae* family also contained peptidases S8 - serine-type endopeptidases, catalyzing the hydrolysis of internal alpha-peptide bonds in the polypeptide chains and containing one FN3 domain without MCRs.

Analysis of the FN3 domains of these proteins using the SAS software demonstrated that only FN3 domains containing annotated MCRs (Group 1) displayed sequence homology with FN3 domains of human proteins [Supplementary Table 4]. Sequences of FN3 domains that lack MCRs showed no homology with FN3 domains of eukaryotic proteins.

## DISCUSSION

In our opinion, studying the FN3 domains of the FN3 protein encoded by the PFNA operon of bifidobacteria is likely to yield important scientific results. Previously, we demonstrated that a fragment of the FN3 proteins of *B. longum* subsp. *longum* GT15 (ΔFN3.1) is capable of selective binding of cytokine TNFα *in vitro*^[24]^. However, since the ΔFN3.1 protein has a heterogeneous domain structure, it is important to determine which region accounts for binding cytokines. To answer this question, we assessed in our work the cytokine-binding ability of two fragments of the ΔFN3.1 protein, which fundamentally differ in their constituent domains: 2D FN3, containing two FN3 domains, and CD FN3, the C-terminal fragment of the FN3 protein [Figure 1]. The recombinant 2D FN3 and CD FN3 proteins were isolated and purified in sufficient quantities to study their binding to TNFα. The structure of one of the protein fragments, namely 2D FN3, was studied using circular dichroism (CD) spectroscopy together with our colleagues from the Moscow State University (Feofanov A.V. - unpublished data). The CD results showed that the antiparallel β-structure predominates in the structure of the studied protein, the formation of which involves 57% of amino acids (including the β-fold). These results are consistent with the data on the organization of typical fibronectin domains of the “β-sandwich” type. We found that neither 2D FN3 nor CD FN3 fragments were able to bind to TNFα separately; only the entire ΔFN3.1 protein exhibited such cytokine-binding properties [Figure 3]. This allowed us to conclude that FN3 domains alone are not enough for binding cytokines. Obviously, the whole ΔFN3.1 protein is required for the formation of the cytokine-binding region. In our future projects, we intend to study the binding that occurs between ΔFN3.1 protein and TNFα, as well as other cytokines using other methods and approaches suited for characterizing protein-protein interaction.

Our hypothesis is also supported by the fact that using the trRosetta servers, we predicted that the ΔFN3.1 protein forms a pocket potentially capable of binding cytokines. The three-dimensional model of the ΔFN3.1 protein structurally resembles the structure of the complex of a eukaryotic protein containing FN3 domains with the GM-CSF cytokine receptor (PDB code 4NKQ)^[34]^. Analysis of the obtained three-dimensional models of the ΔFN3.1, 2D FN3, and CD FN3 proteins shows that only the ΔFN3.1 protein has a V-shaped block in which binding to TNFα can occur, which is consistent with the experimental data. Thus, we experimentally demonstrated that the ΔFN3.1 protein binds TNFα, and by modeling and analyzing the three-dimensional structures of the protein, we predicted its putative binding region. In further experimental work, it is planned to obtain crystal structures of ΔFN3.1 proteins, including those forming a complex with TNFα.

Comparative analysis of amino acid sequences encoding ΔFN3.1 proteins derived from the genomes of sequenced strains of bifidobacteria belonging to the phylotype *B. longum* subsp. *longum*, made it possible to distinguish between four groups of strains. Using the trRosetta software, we demonstrated that these amino acid substitutions are capable of altering the conformation of the spatial structure of the ΔFN3.1 protein, thereby affecting the efficiency and specificity of cytokine binding. Further experimenting and studying of the 3D crystal complex of the ΔFN3.1 bound to TNFα should confirm or refute this hypothesis.

To date, there is no consensus on how FN3 domains originated. It is likely that bacterial FN3 domains were acquired from animals and subsequently spread through horizontal transfer between closely related bacteria^[35]^. However, other studies have suggested that FN3 domains of eukaryotic and archaeal proteins were acquired initially from bacteria^[4,36]^.

Of all the bacterial taxa that we studied, only bifidobacteria harbored proteins of the GH family 3 (beta-glucosidase). In the human body, beta-glucosidase plays an important role in the degradation of glycosphingolipids, which constitute a major part of the cell membrane of eukaryotic cells. It is noteworthy that bifidobacteria [Supplementary Table 1] contained only GH family 3, in which FN3 domains lacked MCRs. Of all the bacterial taxa that we analyzed, GH family 3 (beta-glucosidase) proteins were only present in bifidobacteria. Thus, bifidobacteria, some of the Earth’s oldest inhabitants as well as of anaerobic body cavities of animals, have evolved two types of domains in proteins: (1) FN3 domains encoded by one of the genes of the PFNA operon, which are involved in the interaction with the host’s immune system; (2) FN3 domains embedded in proteins of the GH family 3 capable of cleaving glycosphingolipids (components of the cytoplasmic membrane of human cells), thereby participating in intercellular interactions. Interestingly, strains of the *B. dentium* species living in various teeth cavities of humans possess 12 different GH family 3, which are capable of cleaving cellobiose and salicin^[37]^. Bifidobacteria represent a unique model organism for studying the role of FN3 domains in the interaction with components of the immune system (PFNA operon) and adaptation to environmental niches (GH family 3).

Comparative analysis of the FN3 domains of GH family 3 and the FN3 domain of the protein encoded by the *fn3* gene of the PFNA operon did not reveal any sequence homology between them, which may be grounded in their different evolutionary origins and their involvement in completely different functions: on the one hand, adaptation and usage of various carbohydrate sources abundant in the intestinal microbiota; on the other hand, adaptation to the interaction with components of the host immune system. It is possible that the first group of FN3 domains in hydrolases first emerged in bacteria and then was passed on to eukaryotes, while the second group of FN3 domains (harboring MCRs), such as the one found in the FN3 protein encoded by the *fn3* gene of the bifidobacterial PFNA operon may have been acquired by bifidobacteria from eukaryotes^[4,29,30]^. It is important to note that the PFNA operon in different species of bifidobacteria is localized in different regions of the genome and possibly originated from a common ancestor. The spread of the operon and the ability of the proteins encoded by its genes to interact with the immune system of the host and promote the adaptation of bifidobacteria to various ecological niches led to a high divergence of the operon genes and, possibly, contributed to the speciation of bifidobacteria, the most ancient inhabitants of the Earth and the microbiota of animals.

Elucidation of how “feedback loops” between the human body and commensal microorganisms operate will open up numerous possibilities for regulating the composition and activity of the human microflora, as well as modulating the human immune system.

There is a great interest in the functional role of fibronectin domains containing MCRs in proteins in other taxa of the gut microbiota inhabitants: *Lactobacillacea*, *Bacteroidaceae*, and *Clostridioides*. The possible involvement of such FN3 proteins in the interaction of bacteria with components of the host immune system is an interesting research direction. Type 3 fibronectin domains were found in the families of *Lactobaccilacea*, *Bacteroidaceae,* and *Clostridioides*, mainly in glycoside hydrolases of various families (18, 20, 20b, 31, 43, 65, 92). Depending on the class of GH, FN3 domains were localized in the central part, N-terminal or C-terminal end of the protein [Supplementary Tables 1 and 2]. The location of the FN3 domain and the presence of MCRs may be important indicators of the protein’s function, namely, whether it participates in binding to cytokines and other elements of the immune system. In all analyzed groups of bacteria, FN3 domains seemed to participate in cell adhesion to one or another substrate. In our case, we are particularly interested in the proteins’ capability to interact with the components of the host immune system. For this reason, we focused on proteins containing FN3 domains located in the C-terminal end of the protein and containing MCRs.

MCRs were found in FN3 domains of the GH family 31 in the families *Lactobacillaceae* and *Bacteroidaceae*, in FN3 domains of the GH family 43 in *Bacteroidaceae*, in FN3 domains of the GH family 18, and in FN3 domains of the GH family 20b in *Clostridioides*. Analysis of proteins identified in bacteria of the families *Lactobacillaceae*, *Bacteroidaceae*, and *Clostridioides* revealed that only FN3 domains containing annotated MCRs exhibited sequence homology with FN3 domains of human proteins [Supplementary Table 4]. Sequences of FN3 domains that lacked MCRs exhibited no homology with FN3 domains of eukaryotic proteins. This may indicate the possible involvement of proteins harboring MCRs in interaction with components of the human immune system, namely, by binding to cytokines.

Only the complete ∆FN3.1 protein of *B. longum* subsp. *longum* GT15 can selectively bind to TNFα. FN3 domains alone are not sufficient for binding to TNFα, a C-terminal region is also required. Analysis of 3D models of the 2D FN3, CD FN3, and ΔFN3.1 proteins showed that only the ΔFN3.1 protein is potentially capable of forming a pocket, apparently providing TNFα binding. Bifidobacteria have two types of fibronectin domains - FN3 domains with MCRs, encoded by the PFNA operon, and FN3 domains without MCRs, which can be found in the GHs family 3. Bioinformatics analysis of the genomes of bacteria of the families *Lactobacillaceae*, *Bacteroidaceae,* and *Clostridioides* allowed us to identify proteins containing single FN3 domains. Two groups of FN3 domains were identified in the detected proteins: those containing MCRs and those not having MCRs. Comparative analysis showed that only FN3 domains containing MCRs exhibit homology with FN3 domains of human proteins.

## DECLARATIONS

### Copyright

© The Author(s) 2023.
