# Supplementary material for: Study of the binding of ΔFN3.1 fragments of the Bifidobacterium longum GT15 with TNFα and prevalence of domain-containing proteins in groups of bacteria of the human gut microbiota
Source: Microbiome Res Rep. 2023 Apr 12;2(2):10. doi: 10.20517/mrr.2023.06 (PMC10688814; doi:10.20517/mrr.2023.06)
Supplement: Supplementary file 1 [file mrr-2-2-10-SupplementaryMaterials.pdf]

Supplementary Material

Supplementary Table 1. Proteins harboring FN3 domains that are located outside the PFNA operon in human gut-derived strains of different species of bifidobacteria.

Name of the protein harboring FN3 domains	Species of *Bifidobacterium*	Protein sequence identifier in NCBI	Location of FN3 domain
glycosyl hydrolase family 3	*B. longum* subsp. *longum*	AIW44861	C-terminus, 70 aa
		AIW44860	C-terminus, 71 aa
glycosyl hydrolase family 3	*B. longum* subsp. *infantis*	EEQ54738	Central region, 75 aa
		EEQ55439	C-terminus, 71 aa
glycosyl hydrolase family 3	*B. adolescentis*	BAF40392	Central region, 75 aa
		BAF40391	Central region, 79 aa
glycosyl hydrolase family 3	*B. animalis subsp. lactis*	OQM47627	C-terminus, 71 aa
glycosyl hydrolase family 3	*B. bifidum***	SPU27118	C-terminus, 71 aa
glycosyl hydrolase family 3	*B. breve****	OQM47627	C-terminus, 71 aa
glycosyl hydrolase family 3	*B. angulatum*	AMK57051	C-terminus, 70 aa
		AMK57045	C-terminus, 71 aa
glycosyl hydrolase family 3	*B. dentium*	ADB10806*	C-terminus, 71 aa
glycosyl hydrolase family 3	*B. pseudocatenulatum*	EEG71159	C-terminus, 71 aa

*Apart from the protein ADB10806, there was another 11 proteins belonging to the GH family 3 in the genomes of all *B.dentium* strains: ADB08901, ADB10769, ADB10268, ADB10809, ADB10807, ADB09184, ADB10263, ADB10249, ADB08889, ADB10708, ADB10313.
**Most *B. bifidum* strains do not contain proteins harboring FN3 domains outside the PFNA operon; a single protein of the glycosyl hydrolase family 3 was found in the genome of the strain NCTC10471.
***In *B. breve*, proteins of the glycosyl hydrolase family 3 harboring FN3 domains were present mainly in strains isolated from the gut microbiota of infants.

Supplementary Table 2. Proteins harboring FN3 domains in human gut-derived bacteria of the families *Lactobacillaceae*, *Bacteroidaceae* and *Clostridioides*.

Name of the protein harboring FN3 domains	Species	Protein sequence identifier in NCBI	FN3 domain	
			Location of FN3 domain	Cytokine receptor motif
glycosyl hydrolase family 31	*Lactobacillus acidophilus*	KRK27797	C-terminus, 78 aa	
	Lactobacillus crispatus	QWW29158	C-terminus, 78 aa	
	Lactobacillus gasseri	ABJ59525	C-terminus, 68 aa	
	Lactobacillus helveticus	GFP12355	C-terminus, 78 aa	**VSXWS**
	Lactobacillus paragasseri	EFJ69427	C-terminus, 68 aa	
	Lactobacillus ultunensis	QQP28688	C-terminus, 78 aa	
	Bacteroides fragilis	AKA51120	C-terminus, 73 aa	YSXWA
glycosyl hydrolase family 43	*Bacteroides fragilis*	KXU49890	C-terminus, 86 aa	VSXPS
	Bacteroides ovatus	QUT79180	C-terminus, 85 aa	RSXLS
	Bacteroides uniformis	KAB4109992	C-terminus, 87 aa	VSXRS
		KAB4109856	C-terminus, 80 aa	ENXRS
glycosyl hydrolase family 18	*Clostridium perfringens*	AOY53222	C-terminus, 85 aa	EIXKR
glycosyl hydrolase family 20b		AOY53993	C-terminus, 85 aa	EVXSK
		AOY54134	C-terminus, 85 aa	EIXSK
alpha-N-		AOY53263	C-terminus, 85 aa	ELXSK

acetylglucosaminidase				
sialidase		AOY52943	C-terminus, 85 aa	ELXAK
calcium-binding adhesion protein	*Clostridioides difficile*	QWR70506	Central region, 89 aa	TSXMS
phage tail protein	*Ligilactobacillus salivarius*	RGM22630	N-terminus, 61 aa	ESXKS
porin-like fold protein	*Bacteroides ovatus*	QUT79494	N-terminus, 79 aa	DSXYA
PQQ-binding-like beta-propeller repeat protein		QUT82397	2 FN3 domains - 63 and 80 aa	B FN3(2) - NSXAV
xanthan lyase	*Bacteroides uniformis*	KAB4110324	C-terminus, 87 aa	ESXPS
		KAB4109902	C-terminus, 90 aa	ESXPS
fibronectin type III domain-containing protein (ferrous iron transporter A)	*Lactobacillus acidophilus*	KRK28980	C-terminus, 74 aa	EGXWS
	Lactobacillus helveticus	QAU31920	C-terminus, 74 aa	EGXWS
	Lactobacillus crispatus	QWW29210	C-terminus, 74 aa	TGXWS
		QWW28111	C-terminus, 96 aa	VGXWS
fibronectin type III domain-containing protein	*Phocaeicola dorei*	EEB23863	C-terminus, 92 aa	ESXPS
	Bacteroides fragilis	KXU48500	C-terminus, 92 aa	ESXPS
		KXU42275	C-terminus, 90 aa	ESXPS
		KXU45955	C-terminus, 75 aa	FSXYA
	Bacteroides ovatus	QUT82347	C-terminus, 92 aa	ESXPS

Supplementary Table 3. The occurrence of proteins containing FN3 domains lacking MCRs in bacteria of the families *Lactobacillaceae*, *Bacteroidaceae* and *Clostridioides*.

**Name of the protein harboring FN3 domains**	**Species**	**Protein sequence identifier in NCBI**	**Location of FN3 domain**
peptidase S8	*Lactobacillus acidophilus*	KRK28186	Central region
	Lacticaseibacillus casei	KLI75633	C-terminus
	Lacticaseibacillus paracasei	QDP35932	Central region
		QDP37060	
		QDP35931	
	Lactobacillus helveticus	GFP12550	Central region
		GFP12480	
		GFP12998	
	Lactiplantibacillus plantarum	TAR98505	C-terminus
	Lacticaseibacillus rhamnosus	KFC34860	C-terminus
		KFC37136	Central region
	Ligilactobacillus ruminis	TGJ61417	Central region
	Lactobacillus ultunensis	QQP27945	Central region
glycosyl hydrolase family 3	*Lacticaseibacillus casei*	KLI75030	Central region
glycosyl hydrolase family 20	*Phocaeicola dorei*	EEB26793	Central region
		EEB26209	Central region
		EEB26053	Central region
		EEB24908	Central region
		EEB24073	Central region
		EEB25570	Central region
glycosyl hydrolase family 20	*Bacteroides fragilis*	KXU48352	Central region
		KXU46540	Central region
		KXU42591	Central region

glycosyl hydrolase family 20	*Bacteroides ovatus*	QUT83024	Central region
		QUT82175	Central region
		QUT81276	Central region
		QUT80688	Central region
		QUT79064	Central region
glycosyl hydrolase family 20	*Bacteroides uniformis*	KAB4108785	Central region
		KAB4105426	Central region
glycosyl hydrolase family 20	*Phocaeicola vulgatus*	KAB6712571	Central region
		KAB6712466	Central region
		KAB6712388	Central region
		KAB6712252	Central region
		KAB6708280	Central region
glycosyl hydrolase family 92	*Bacteroides uniformis*	KAB4108790	Central region
glycosyl hydrolase family 65	*Clostridium perfringens*	AOY54643	Central region
glycosyl hydrolase family 3	*Clostridiales bacterium* (human gut metagenome)	PWL55319	Central region
SGNH_hydrolase		PWL49978	Central region

Supplementary Table 4. Sequence homology between FN3 domains of the identified proteins and FN3 domains of human proteins.

Name of the protein harboring FN3 domains	Species	Protein sequence identifier in NCBI	Homologous 3D Structures according to SAS Program		
			% of homology	Name of the structure	Number in PDB
glycosyl hydrolase family 31	*L. acidophilus*	KRK27797	28,2	Solution structure of the second fibronectin type III domain of human kiaa1568 protein	**1ujt**
	L. crispatus	QWW29158			
	L. helveticus	GFP12355			
	L. ultunensis	QQP28688			
	L. gasseri	ABJ59525	31,3	The solution structure of the second fibronectin type III domain of human neogenin	**1x5g**
	L. paragasseri	EFJ69427			
	B. fragilis	AKA51120	28,8	Solution structures of the fn3 domain of human tripartite motif protein 9	**2db8**
glycosyl hydrolase family 43	*B. fragilis*	KXU49890	27,2	The solution structure of the sixth fibronectin type III domain of human neogenin	**1x5k**
	B. ovatus	QUT79180			
	B. uniformis	KAB4109992	25,0		
		KAB4109856	31,0		
glycosyl hydrolase family 18	*Clostridium perfringens**	AOY53222	31,2	Titin module a71 from human cardiac muscle, nmr, 50 structures	**1bpv**
glycosyl hydrolase family 20b		AOY53993<br>AOY54134	28,4<br>31,8	The solution structure of the second fibronectin type III domain of human neogenin	**1x5g**
alpha-N-acetylglucosaminidase		AOY53263	28,1	Solution structure of the second fibronectin type III domain of human netrin receptor dcc	**2ed8**
sialidase		AOY52943	23,9	The solution structure of the second fibronectin type III domain of human neogenin	**1x5g**
calcium-binding adhesion protein	*Clostridioides difficile*	QWR70506	33,3		
phage tail protein	*L. salivarius*	RGM22630	42,9	Solution structure of the fibronectin type-III domain of human fibronectin type III	**1x5x**

				domain containing protein 3	
porin-like fold protein	*B. ovatus*	QUT79494	36,8	Collagen alpha-1(vii) chain	**A26A**
PQQ-binding-like beta-propeller repeat protein		QUT82397	26,1	Structure of a fragment of human fibronectin containing the 10th, 11th and 12th type III domains	**6xay**
xanthan lyase	*B. uniformis*	KAB4110324	30,4	Solution structure of the first fibronectin type iii domain of human kiaa0343 protein	**1uey**
		KAB4109902	28,7	Solution structure of the second fibronectin type iii domain of human netrin receptor dcc	**2ed8**
fibronectin type III domain-containing protein (ferrous iron transporter A)	*L. acidophilus*	KRK28980*	26,9	Collagen alpha-1(vii) chain	**A26A**
	L. helveticus	QAU31920*	30,3		
	L. crispatus	QWW29210	26,0	Crystal structure of the tenth type iii cell adhesion module of human fibronectin	**1fna**
		QWW28111	30,6	Solution structure of the second fibronectin type III domain of human kiaa1568 protein	**1ujt**
fibronectin type III domain-containing protein	*Phocaeicola dorei*	EEB23863	34,6	Solution structure of the first fibronectin type iii domain of human kiaa0343 protein	**1uey**
	Bacteroides fragilis	KXU48500	31,6	Crystal structure of the titin a-band domain a3	**4o00**
		KXU45955	31,2	Solution structures of the fn3 domain of human receptor-type tyrosine- protein phosphatase f	**2edy**
		KXU42275	27,5	Solution structure of the first fibronectin type iii domain of human kiaa0343 protein	**1uey**
	Bacteroides ovatus	QUT82347	30,9		

KRK28980* and QAU31920* - these proteins also exhibit 31-33% sequence homology with the 3D structure of 6rpx Cytokine receptor-like factor 3 c-terminus residues (Mus musculus).

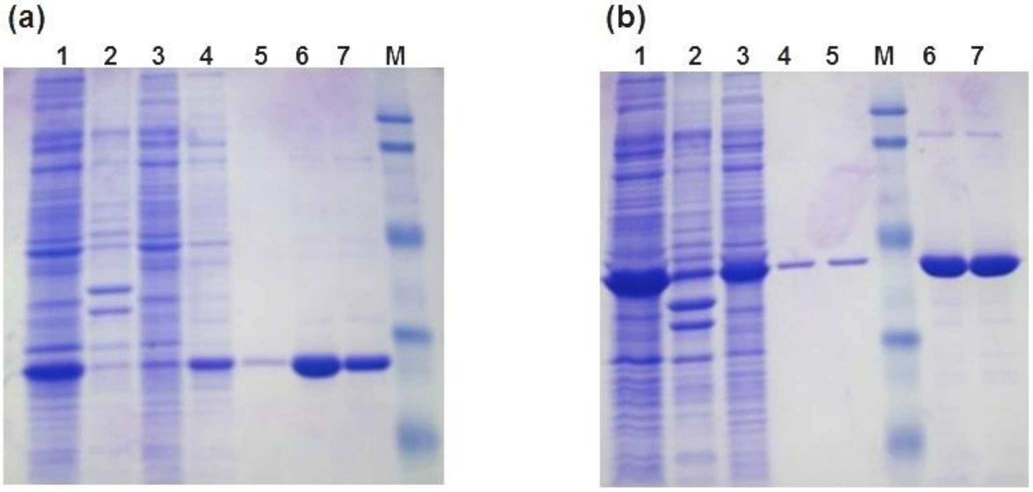

Supplementary Figure 1. Electrophoregram of stepwise of purification on Ni-NTA Superflow of proteins 2D FN3 (a) и CD FN3 (b). Lanes: 1 – lysate, 2 – pellet, 3 – flow-through, 4 – wash_1; 5 – wash_2; 6 – eluate_1; 7 – eluate_2; M – molecular weight protein marker SM0441 (Fermentas, Lithuania).

```
GT15        PEPPLLSPVAGEPQDSAVSLTWTPGAANGSPITNYKVSWTGAATGEKDCGAVTSCQVTGL  60
MCC10031    PEPPLLSPVAGEPQDSAVSLTWTPGAANGSPITNYKVSWTGAVTGEKDCGAVTSCQVTGL  60
MCC10014    PEPPLLSPVAGEPQDSAVSLTWTPGAANGSPITNYKVSWTGAVTGEKDCGTVTSCQVTGL  60
MCC10099    PEPPLLSPVAGEPQDSAVSLTWTPGAANGSPITNYKVSWTGAATGEKDCGAVTSCQVTGL  60
            ******************************************.*******:*********

GT15        KNGKTYSFTVAARNDVGWSKPSTAVEATPDKVPSAPTDVTVTGGNKTAKVTWKAPSGDFS  120
MCC10031    KNGKTYSFTVAARNDVGWSKPSTAVEATPDKVPSAPTDVTVTGGNKTAKVTWKAPSGDFS  120
MCC10014    KNGKTYSFTVAARNDVGWSKPSTAVEATPDKVPSAPTDVTVTGGNKTAKVTWKAPSGDFS  120
MCC10099    KNGKTYSFTVAARNDVGWSKPSTAVEATPDKVPSAPTDVTVTGGNKTAKVIWKAPSGDFS  120
            ************************************************** *********

GT15        AVDNYSVTVTGAGAPQTKETGNTATELSFTFNNNDISDGTAITATVKAHNKINWSAESAA  180
MCC10031    AVDNYSVTVTGAGAPQTKETGNTATELSFTFNNNDISDGTAITATVKAHNKINWSAESAA  180
MCC10014    AVDNYSVTVTGAGAPQTKETGNTATELSFTFNNNDISDGTAITATVKAHNKINWSAESAA  180
MCC10099    AVDNYSVTVTGAGAPQTKETGNTATELSFTFNNNDISDGTAITATVKAHNKINWSAESAA  180
            ************************************************************

GT15        SPSEKIWGDPDAPNIALSNDDTTVTAKVTLGNNRNAGCRRISLGGDVKDTIDCSDSGATF  240
MCC10031    SPSEKIWGDPDAPNIALSNDDTTVTAKVTLGNNRNAGCRRISLGGDVKDTIDCSDSGATF  240
MCC10014    SPSEKIWGDPDAPNIALSNDDTTVTAKVTLGNNRNAGCRRISLGGDVKDTIDCSDSGATF  240
MCC10099    SPSEKIWGDPDAPNIALSNDDTTVTAKVTLGNNRNAGCRRISLGGDVKDTIDCSDSGATF  240
            ************************************************************

GT15        DINENDLNTREITVTATVVPQRDASSGTGKSSFVPQYKVQPPTDVYTTGSGSTCTVHWTK  300
MCC10031    DINENDLNTREITVTATVVPQRDASSGTGKSSFVPQYKVQPPTDVYTTGSGSTCTVHWTK  300
MCC10014    DINENDLNTREITVTATVVPQRDASSGTGKSSFVPQYKVQPPTDVYTTGSGSTCTVHWTK  300
MCC10099    DINENDLNTREITVTATVVPQRDASSGTGKSSFVPQYKVQPPTDVYTTGSGSTCTVHWTK  300
            ************************************************************

GT15        QGHAQSYSVTADGIDSGTATNRNSLDFRMSPWAKCNTASVQQVFNGATSDAVTGGPHNGA  360
MCC10031    QGHAQSYSVTADGIDSGTATNRNSLDFRMSPWAKCNTASVQQVFNGATSDAVTGGPHNGA  360
MCC10014    QGHAQSYSVTADGIDSGTATNRNSLDFRMSPWAKCNTASVQQVFNGATSDAVTGGPHNGA  360
MCC10099    QGHAQSYSVTADGIDSGTATNRNSLDFRMSPWAKCNTASVQQVFNGATSDAVTGGPHNGA  360
            ************************************************************

GT15        PYTYEKKAVINKPQELRWSDNAHVINVTGGSVDVYGKSATVQIIINGQPFTWNSGQPLDV  420
MCC10031    PYTYEKKAVINKPQELRWSDNAHVINVTGGSVDVYGKSATVQIIINGQPFTWNSGQPLDV  420
MCC10014    PYTYEKKAVINKPQELRWSDNAHVINVTGGSVDVYGKSATVQIIINGQPFTWNSGQQLDV  420
MCC10099    PYTYEKKAVINKPQELRWSDNAHVINVTGGSVDVYGKSATVQIIINGQPFTWNSGQQLDV  420
            ******************************************************** ***

GT15        TDLAVAADGNYAWSVKVTGGPGYSGLDNTADGGSVAGTRPSDSSEGTKSLKSGTDVTTLA  480
MCC10031    TDLAVAADGNYAWSVKVTGGPGYSGLDNTADGGSVAGTRPSDSSEGTKSLKSGTDVTTLA  480
MCC10014    TDLTVAADGNYAWSVKVTGGPGYSGLDNTADGGSVAGTRPSDSSEGTKSLKSGTDVTTLA  480
MCC10099    TDLTVAADGNYAWSVKVTGGPGYSGLDNTADGGSVAGTRPSDSSEGTKSLKSGTDVTTLA  480
            ***:********************************************************

GT15        KNPWILGFGNTPISSTTIHKQ        501
MCC10031    KNPWILGFGNTPISSTTIHKQ        501
MCC10014    KNPWILGFGNTPISSTTIHKQ        501
MCC10099    KNPWILGFGNTPISSTTIHKQ        501
            *********************
```

Supplementary Figure 2. Alignment of amino acid sequences of ΔFN3.1 proteins from *B. longum* subsp. *longum* GT15 (Group 1), MCC10031 (Group 2), MCC10014 (Group 3), and MCC10099 (Group 4). Amino acid substitutions are highlighted in black.
